# Retroviral Vectors for Analysis of Viral Mutagenesis and Recombination

**DOI:** 10.3390/v6093612

**Published:** 2014-09-24

**Authors:** Jonathan M.O. Rawson, Louis M. Mansky

**Affiliations:** 1 Institute for Molecular Virology, University of Minnesota, Moos Tower 18-242, 515 Delaware St SE, Minneapolis, MN 55455, USA; E-Mail: rawso018@umn.edu; 2 Molecular, Cellular, Developmental Biology & Genetics Graduate Program, University of Minnesota, 6-160 Jackson Hall, 321 Church St SE, Minneapolis, MN 55455, USA; 3 Department of Diagnostic and Biological Sciences, School of Dentistry, University of Minnesota, 515 Delaware St SE, Minneapolis, MN 55455, USA; 4 Department of Microbiology, University of Minnesota, 420 Delaware St SE, Minneapolis, MN 55455, USA

**Keywords:** retrovirus, lentivirus, reverse transcription, evolution, mutation, recombination, retroviral vector

## Abstract

Retrovirus population diversity within infected hosts is commonly high due in part to elevated rates of replication, mutation, and recombination. This high genetic diversity often complicates the development of effective diagnostics, vaccines, and antiviral drugs. This review highlights the diverse vectors and approaches that have been used to examine mutation and recombination in retroviruses. Retroviral vectors for these purposes can broadly be divided into two categories: those that utilize reporter genes as mutation or recombination targets and those that utilize viral genes as targets of mutation or recombination. Reporter gene vectors greatly facilitate the detection, quantification, and characterization of mutants and/or recombinants, but may not fully recapitulate the patterns of mutagenesis or recombination observed in native viral gene sequences. In contrast, the detection of mutations or recombination events directly in viral genes is more biologically relevant but also typically more challenging and inefficient. We will highlight the advantages and disadvantages of the various vectors and approaches used as well as propose ways in which they could be improved.

## 1. Introduction

Retroviruses share a remarkable capacity to rapidly evolve, which can be attributed to several key variables: high rates of mutation, recombination, and replication, large numbers of infected cells, and strong positive selective pressures [1,2,3]. These factors act together to drive the expansion of a small number of viruses that are initially transmitted to a particular host into a population of diverse but interacting variants, termed the viral quasispecies. Genetic diversification enables escape from the host immune response, accelerates the emergence of drug resistance, and promotes cross-species transmission. The ability of human retroviruses like human immunodeficiency virus type-1 (HIV-1) to rapidly evolve poses a tremendous challenge to the development of effective prophylactics and therapeutics.

Retroviruses mutate at an average rate of ~3 × 10^−5^ mutations/base pair (bp)/cycle, corresponding to roughly one mutation per three genomes synthesized [4,5,6,7,8]. This mutation rate is ~10–1000 fold higher than DNA viruses (10^−6^–10^−8^ mutations/bp) [9] and at least 10,000-fold higher than for eukaryotic DNA (≤10^−9^ mutations/bp) [10]. Mutations could arise during several different steps of the retroviral life cycle. Reverse transcriptase (RT) converts the single-stranded viral RNA into double-stranded DNA and is thought to be one of the key drivers of viral mutagenesis, primarily due to its high error rates (typically 10^−4^–10^−5^ mutations/bp) [11] *in vitro*. However, RNA polymerase II (Pol II) can also generate mutations when transcribing the viral genomic RNA from the integrated proviral DNA [12], though the relative contribution of Pol II compared to RT has not been well defined. Cellular DNA polymerases can also create mutations when replicating the integrated provirus during cell division, but the high fidelity of cellular DNA replication argues that this is a relatively minor source of virus variation. Lastly, nucleic acid-editing enzymes such as the APOBEC3 family of cytosine deaminases can edit minus strand viral DNA during reverse transcription [13,14], ultimately leading to G-to-A mutations on the plus strand viral DNA. APOBEC3-mediated editing is often lethal, as multiple G-to-A mutations are usually induced in the same provirus (*i.e.*, G-to-A hypermutation), but editing has been reported to accelerate viral evolution in certain contexts [15,16]. Furthermore, while HIV-1 expresses an accessory protein called Vif to counteract APOBEC3 proteins, Vif alleles have been shown to vary widely in their abilities to counteract various APOBEC3 proteins [17,18,19]. In addition, G-to-A hypermutants have often been observed in patient samples [17,20,21,22,23,24,25,26], which provides strong evidence that APOBEC3-mediated editing occurs *in vivo*. All of these processes together contribute to the high mutation rates of retroviruses, leading to the evolution of variants that may confer drug resistance, improve transmissibility, or allow for cytotoxic T-cell or neutralizing antibody escape. Many additional variables have been shown to influence retroviral mutagenesis, including RT variants [27,28,29,30,31], antiviral drugs [27,28,30,32], and accessory proteins [28,33,34,35]. 

In addition to mutating at high rates, retroviruses are also able to recombine at high rates (e.g., ~3–14 crossovers/genome/cycle for HIV-1) [36,37,38,39,40,41,42]. Retroviruses are able to recombine rapidly because (unlike other RNA viruses) they co-package two copies of the RNA genome into every viral particle. During reverse transcription, recombination between RNA genomes often occurs but only one DNA provirus is ultimately formed, such that retroviruses are considered pseudodiploid in nature [43]. Recombination events between identical co-packaged genomes are effectively silent since new viral sequences are not generated. In contrast, recombination events between distinct genomes (*i.e.*, from heterozygous virions) can lead to novel viral variants. Recombination permits the completion of DNA synthesis even in the presence of RNA damage and also promotes the generation of novel variants through the shuffling of viral mutations. For example, recombination can link beneficial mutations together, such as low-level drug resistance mutations into a highly drug-resistant complex [44] or single-drug resistance mutations into multi-drug resistance [45]. Conversely, recombination can allow escape from deleterious or lethal mutations [46,47,48]. While often beneficial, recombination can also dissociate co-adapted mutations, leading to unfit viral variants [49]. The importance of recombination to the establishment of the global AIDS pandemic is clear: 65 circulating recombinant forms (CRFs) have been identified to date [50], accounting for ~20% of HIV-1 infections worldwide [51].

As a pre-requisite for recombination to occur between distinct viruses, the producer cells must become dually infected, either as a result of co-infection (near simultaneous infection) or super-infection (sequential infection). Thus, factors that alter the incidence of dual infection through either mechanism will impact the frequency of recombination. HIV-1 readily co-infects primary CD4^+^ T-cells *ex vivo* with little evidence of interference [52,53,54,55]. In fact, co-infection has been found to occur more frequently than expected from random interactions between viruses and cells [52,53], but this may be due to reactivation of silent proviruses upon co-infection [56]. In contrast to co-infection, cells infected by HIV-1 (and many other retroviruses) are resistant to re-infection, a phenomenon called super-infection resistance [57]. Super-infection of humans at the organismal level has often been documented [58,59,60,61,62,63], though initial infection may be somewhat protective of re-infection [62]. However, such individuals do not necessarily contain super-infected cells, as only a small fraction of CD4^+^ T-cells becomes infected. Nonetheless, the wide prevalence of intra-subtype, inter-subtype, and inter-group recombinants of HIV-1 demonstrates that cellular super-infection resistance is not absolute. Of note, dually infected splenocytes can readily be observed in samples from HIV-1 infected individuals [64,65] or SIV_mac_-infected rhesus macaques [66]. However, another study found that most peripheral blood CD4^+^ T-cells harbor only a single provirus [67]. Unfortunately, it is not yet clear whether these discrepancies are due to the different compartments sampled, unique features of the infected individuals, or other factors. 

In addition to the incidence of dual infection, rates of retroviral recombination are influenced by factors that alter the ability of genetically distinct genomes to co-package into the same virus particle. For example, the HIV-1 genome contains the dimerization initiation signal (DIS), a six nucleotide palindromic sequence within the first stem loop of the 5’ untranslated region. The DIS sequence is the dominant factor that drives HIV-1 genomic RNA co-dimerization and co-packaging [68,69,70,71]. Thus, HIV-1 variants with matched DIS sequences ultimately recombine much more frequently than those with mismatched DIS sequences. Additionally, the rate of recombination depends on the frequency of template switching during reverse transcription. Template switching is thought to be controlled by the relative balance of polymerase and RNase H activities of RT, referred to as the dynamic copy choice model of recombination [72,73]. Factors that reduce the speed of RT-mediated DNA synthesis promote template switching, including low dNTP pool levels [72,74,75,76], RNA secondary structures [77,78,79], and mutations in RT that impair processivity [72,75,76]. In contrast, RT mutations that reduce RNase H activity decrease the level of template switching [72,75,80]. The level of template switching also correlates fairly well with the degree of sequence homology between templates, such that closely related sequences (e.g., from the same subtype of HIV-1) recombine more frequently than divergent sequences (e.g., from different subtypes of HIV-1) [71,81]. 

In developed countries, the advent of highly active antiretroviral therapy (HAART) directed against HIV-1 has enabled the indefinite suppression of viral replication in the vast majority (>95%) of infected individuals, provided proper drug adherence is maintained. This raises the issue of the settings in which the high mutation and recombination rates of HIV-1 remain relevant. Prior to the initiation of treatment, rapid viral replication and diversification permit escape from CD8^+^ cytotoxic T-cell and neutralizing antibody responses, ultimately preventing effective immune system control of the virus. Defining the roles of viral mutation and recombination in these processes may inform efforts to develop an effective vaccine. Further, during HAART, a low level of ongoing viral replication may persist in areas of limited drug penetration, such as the gut-associated lymphoid tissue and central nervous system [82,83], permitting continual viral evolution. However, the presence of ongoing viral replication during HAART has remained controversial, and the predominant mechanism of viral persistence is thought to be latent infection of resting CD4^+^ T-cells [84]. HAART is also not completely suppressive in all individuals, as ~3% of infected individuals develop triple-class virological failure [85], though this may result from suboptimal drug adherence. Additionally, high rates of mutation and recombination are features often shared by other RNA viruses [9,86,87,88], many of which cannot be effectively countered by drug treatment. The investigation of these processes in HIV-1 will serve as a useful model system for studying the genetic diversification of other RNA viruses. 

Although many advances have been made, there are still a number of fundamental questions surrounding the nature of retroviral mutagenesis and recombination that remain unanswered. For example, very few studies have examined these processes in primary cells rather than immortalized cell lines. Differences in dNTP pool levels and expression of relevant cellular factors between immortalized cell lines and primary cells could have a large impact on viral mutagenesis and recombination. Even primary cell types can differ substantially in this regard, as the most relevant targets of HIV-1 infection, activated CD4^+^ T-cells and macrophages, differ by ~130–250-fold in dNTP pool levels [89]. In addition, very few studies have examined mutagenesis or recombination in native viral genes rather than foreign reporter genes, primarily due to the increased difficulty of detecting such events. Given this, there remains a clear need for continued dissection of these processes in retroviruses, and improved vectors and methodologies to aid in these investigations. 

Many different vectors and approaches have been used to examine retroviral mutagenesis and recombination. Retroviral vectors can broadly be divided into two categories: those that utilize reporter genes (e.g., antibiotic resistance genes, β-galactosidase, or fluorescent proteins) and those that utilize viral genes as targets for mutation or recombination. Reporter gene vectors greatly facilitate the detection, quantification, and characterization of mutants or recombinants, but the results may not be representative of native viral genes in regards to the frequency or types of mutation and/or recombination hotspots. In contrast, the detection of mutations or recombination events directly in viral genes is more biologically relevant, but the methods to do so are also much less efficient. Most of the retroviral vectors utilized for studies of viral mutagenesis and recombination are not replication-competent and must be trans-complemented in order to produce infectious vector virus. The benefit of this approach is that it allows for the more accurate quantification of mutation and recombination rates, as all events must occur in a single cycle of replication. In other instances, replication-competent vectors have been used to examine retroviral mutagenesis and recombination, but they have been limited to a single round of replication by neutralizing antibodies or antiviral drugs. In contrast, multi-cycle replication assays are valuable for understanding how natural selection acts upon emerging mutants and recombinants, and some of these studies will briefly be addressed. Here, the design and application of a number of different retroviral vectors that have been employed in assays to investigate retroviral mutagenesis and recombination is reviewed. The benefits and drawbacks of each approach are highlighted and potential ways to improve current vectors and methodologies are explored. 

## 2. Vectors for Examination of Retroviral Mutagenesis

### 2.1. Reporter Gene Vectors for Reversion Assays

**Figure 1:**
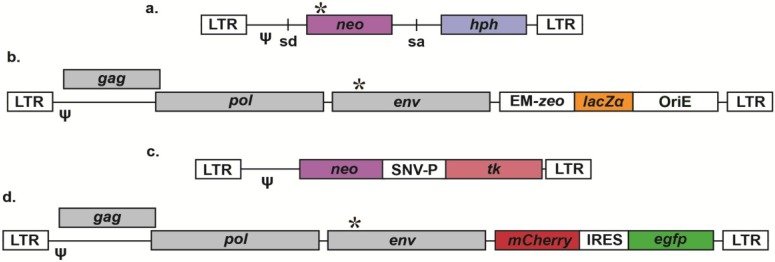
Vectors for the examination of retroviral mutagenesis. Most accessory genes and cis elements are not shown for simplicity; * represents inactivating mutations, while ψ represents packaging signals. (**a**) SNV vector to detect mutations that restore neo, conferring G-418 resistance [90]; sd = splice donor, sa = splice acceptor. (**b**) HIV-1 vector that detects mutations in *lacZα* that prevent complementation and expression of functional β-galactosidase in E. coli, ultimately resulting in white bacterial colonies in the presence of X-gal [101]; EM = bacterial promoter, *zeo* = zeomycin resistance gene, OriE = E. coli origin of replication. (**c**) MLV vector to monitor mutations that eliminate thymidine kinase (*tk*), granting resistance to the thymidine analog bromodeoxyuridine (BrdU) [8]; SNV-P = SNV promoter (U3). (**d**) HIV-1 vector to detect mutations that eliminate expression of mCherry or enhanced green fluorescent protein (EGFP) by flow cytometry [107]; IRES = internal ribosome entry site.

Some of the earliest vectors for quantification of retroviral mutation rates relied upon the detection of mutations that restored defective drug resistance genes (see Table 1). Many of these initial studies utilized spleen necrosis virus (SNV)-based vectors. SNV is an avian gammaretrovirus capable of infecting several types of mammalian cells and has often been employed as a model system for retrovirus biology. The first mutation rate of a retrovirus was determined using a SNV-based vector containing a dysfunctional neomycin resistance gene (*neo*) and a functional hygromycin B resistance gene (*hph*), as shown in Figure 1A [90]. The neomycin resistance gene had a single point mutation introducing a premature stop codon, and *neo* revertants could be selected for using the neomycin analog G-418. The reversion mutation frequency of SNV was determined by calculating the ratio of G-418-resistant (neo^r^) to hygromycin-resistant (hygro^r^) colonies and was found to be 2.2 × 10^−5^. In this particular assay, the reversion mutation frequency was equivalent to the reversion mutation rate (defined as mutations/bp/cycle), as the size of the mutational target was a single base and the vector was limited to one round of replication.

Viral mutagenesis has been investigated in several other retroviruses using reversion vectors as well. The mutation rate of murine leukemia virus (MLV), another gammaretrovirus, has been determined using a *neo/hph* cassette similar to the one used for SNV [91]. More recently, the mutagenesis of HIV-1 has been investigated using a defective luciferase gene containing an insertion of eight T residues near the 5’ end of the gene [30]. This vector was designed specifically to detect frameshift events, which are often triggered by homopolymeric runs [92,93,94,95]. Relative mutation frequencies were determined by comparing ratios of relative light units to the viral titers, which were determined using an integrated LTR-driven marker gene in the same cell line. In addition to luciferase, vectors containing an inactivated blasticidin resistance gene (*bsr*) and enhanced yellow fluorescent protein (EYFP) have been used to measure mutation rates of several retroviruses, including avian leukosis virus (ALV), MLV, and HIV-1 [96]. In these assays, reversion frequencies were defined as the ratio of blasticidin-resistant/EYFP^+^ cells to all EYFP^+^ cells. 

**Table 1:** Targets for analysis of retroviral mutagenesis.

Target^1^	Assay Type^2^	Virus	References
***neo***	Reporter (R)	SNV, MLV	[90,91]
***tk***	Reporter (F)	MLV, HIV-1	[8,97,98]
***lacZα*/*lacZ***	Reporter (F)	SNV, BLV, HTLV-1, HIV-1	[4,5,6,7,27,28,29,31,32,33,34,35,99,100,101,102]
***luc***	Reporter (R)	HIV-1	[30]
***bsr***	Reporter (R)	ALV, MLV, HIV-1	[96]
***hsa***	Reporter (F)	HIV-1	[103,104]
***egfp***	Reporter (F)	HIV-1	[105,106,107,108]
**Viral**	HTA	RSV	[109]
	RNase T_1_	MLV	[110]
	SSCP	HIV-1	[12]
	Sequencing (Sanger)	HIV-1	[111,112]
	Sequencing (NGS)	HIV-1	[40]

^1^ Reporter gene targets include antibiotic resistance genes (*neo*, *bsr*), thymidine kinase (*tk*), β-galactosidase (*lacZα* or full-length *lacZ*), the cell surface marker heat stable antigen (*hsa*), and enhanced green fluorescent protein (*egfp*); ^2^Reporter gene targets are included for both reversion assays (R) as well as forward mutational assays (F). Assays that can detect mutations directly in viral genes include heteroduplex tracking (HTA), RNase T_1_ fingerprinting, single-strand conformation polymorphisms (SSCP), Sanger sequencing, and next-generation sequencing (NGS) technologies.

Although used infrequently, reversion reporter vectors do greatly facilitate the detection and quantification of viral mutants, and sequencing can determine the nature of reverting mutations. However, these vectors can only detect the few specific mutations that are able to restore a functional gene phenotype. Thus, many types of mutations cannot be detected at all, and those mutations that can be observed are limited to very narrow sequence contexts. Therefore, calculated mutation rates may not be representative of the overall mutation rate within the reporter gene or virus. In addition, the influence of factors on the mutation spectra cannot readily be addressed. For these reasons, vectors that instead rely on the elimination of functional reporter genes (*i.e.*, forward mutation assays) have been utilized much more frequently than vectors that measure reversion mutation frequencies.

### 2.2. Reporter Gene Vectors for Forward Mutational Assays

#### 2.2.1. LacZα/LacZ (β-galactosidase)

Rather than monitoring the restoration of defective reporter genes, forward mutational assays detect the elimination of functional reporter gene products. Thus, these assays can detect every mutational class, including the twelve types of substitutions, insertions, deletions, and more complex events. Furthermore, mutations can be detected in a variety of sequence contexts. Though many different reporter genes have been used in these assays (see Table 1), the lacZα peptide gene (or, in some cases, the full-length lacZ) is one of the most widely used reporters for analyses of retroviral mutagenesis. The lacZα gene encodes a small fragment (~150–170 bp) of β-galactosidase that can interact with another fragment (lacZω) to reconstitute full-length β-galactosidase. The formation of functional β-galactosidase can be detected by blue-white color screening in E. coli. This system has been used to determine forward mutation rates and spectra for a number of different retroviruses (see Table 1). Furthermore, this system has been used to characterize the impact of factors such as drugs, drug resistance-associated mutations, and viral accessory proteins on viral mutagenesis.

While specific details vary between laboratories, the general steps of the lacZα assay are as follows: First, producer cells are established that contain an integrated packaging construct with a shuttle cassette. The shuttle cassette often consists of lacZα, an antibiotic resistance gene (for selection of producer and target cells), and a bacterial origin of replication (see Figure 1B). Virus production is initiated by transient transfection with helper plasmids, and the collected virus is used to infect target cells. The virus can only replicate a single time in the target cells due to the lack of essential viral genes. Next, the lacZα DNA is recovered, usually through a process that avoids the use of PCR, as PCR can generate background errors. For example, the lacZα DNA can be recovered by purification with the Lac repressor protein [4] or by Hirt extraction [101], which recovers extrachromosomal DNA. For Hirt extraction, vectors have been designed with inactivating mutations in integrase to facilitate the recovery of lacZα DNAs. The DNA can then be transformed into bacteria, selected with antibiotics, and subjected to blue-white color screening using the chromogenic substrate X-gal. Mutation frequencies are calculated by dividing the number of mutant colonies (white or light blue) by the total number of colonies. Forward mutation rates can be calculated by dividing the mutation frequencies by the size of lacZα or by the known number of detectable sites [113,114] for mutations in lacZα (this is possible because lacZα has been extensively mutated). Mutation spectra can be determined by targeted sequencing of white colonies, leading to identification of a wide array of mutations. 

#### 2.2.2. Thymidine Kinase (TK)

In addition to lacZα, the herpes simplex virus thymidine kinase (tk) gene has been used in forward reporter vectors in analyses of retroviral mutagenesis (Table 1). Thymidine kinase is a nucleotide salvage enzyme that converts thymidine into thymidine monophosphate (TMP). Cells expressing thymidine kinase are resistant to inhibitors of *de novo* TMP synthesis (e.g., aminopterin) but sensitive to the thymidine analog bromodeoxyuridine (BrdU). These vectors contain both tk as well as an antibiotic resistance gene (hygro or neo) for determination of viral titers (see Figure 1C). To use these vectors, producer cells are established, virus production is initiated by transfection of helper plasmids, and TK^-^ target cells are infected, such that the viral vector is the only source of TK. Forward mutation frequencies can be calculated by dividing the number of antibiotic-resistant (hygro^r^ or neo^r^) and BrdU-resistant (BrdU^r^) colonies by all antibiotic-resistant colonies. Forward mutation rates can be calculated by dividing mutation frequencies by the size of tk (~1.2 kb), and spectra can be examined by PCR and sequencing. Relative to lacZα, tk vectors offer several advantages, such as the ability to propagate and further characterize mutant proviruses that survive drug selection. As tk is substantially longer than lacZα (~1.2 kb and ~0.2 kb, respectively), the general likelihood of observing mutations is greater. However, the increased length of tk also necessitates that more sequencing be performed to identify all mutations within the reporter gene. In addition, the use of PCR to recover tk sequences results in some level of background errors. Further, many types of cells cannot be used as target cells of infection in this assay because they express endogenous TK.

#### 2.2.3. Fluorescent Proteins and Cell Surface Markers

HIV-1 mutagenesis has been investigated using several single-cycle reporter vectors that encode fluorescent proteins and/or cell surface markers, which can be stained by antibodies conjugated to fluorescent dyes. To construct the first vector [105], the mouse heat stable antigen (hsa, a cell surface marker) and enhanced green fluorescent protein (egfp) were inserted in place of nef within an HIV-1 envelope-deficient molecular clone. The reporter genes were separated by an internal ribosome entry site (IRES) element necessary for translation of EGFP. More recently, the hsa gene has been replaced with mCherry (Figure 1D), eliminating the need for antibody staining [107]. In addition, a similar mCherry/egfp vector has been constructed for mutagenesis studies in human immunodeficiency virus type-2 (HIV-2) [115]. In order to determine mutant frequencies, these vectors are co-transfected with an envelope plasmid into 293T cells. Viral stocks are collected and used to infect target cells, and flow cytometry is performed to quantify the number of infected cells (cells expressing at least one reporter) as well as the number of cells with mutant proviruses (cells expressing only one reporter). Mutant frequencies are calculated by dividing the number of mutants by the total number of infected cells. To assess mutational spectra, fluorescence-activated cell sorting (FACS) can be performed to isolate cells expressing only one reporter gene, from which genomic DNA can be purified. Lastly, the defective gene (e.g., egfp from mCherry^+^/EGFP^−^ cells) can be amplified by PCR, ligated into a cloning vector, and sequenced. To date, these vectors have been employed to examine the impact of small molecule mutagens [105,106,107,108,112,115,116], drug resistance-associated mutations [117], cell type [104], and APOBEC3G [103,108] on HIV-1 mutagenesis. The primary advantage of these vectors is that they allow for rapid determination of infectivities and mutant frequencies, and thus are ideally suited for the screening of small molecules for antiviral and/or mutagenic activities. However, cell sorting is required to enrich for mutant sequences prior to further processing, as the vast majority of sequences are wild-type in the absence of sorting. Further, PCR is required to amplify, clone, and sequence defective genes. Some mutations that eliminate expression of EGFP may occur within the IRES element necessary for EGFP translation, such that the entire IRES-EGFP region (~1.3 kb) must be sequenced to identify all responsible mutations. 

### 2.3. Detection of Mutations in Viral Genes

#### 2.3.1. Biochemical and Sanger Sequencing-Based Approaches

Rather than detecting mutations in reporter genes, some studies have assessed viral mutagenesis by directly detecting mutations in viral genes (Table 1). As reporter genes are unnecessary for this approach, a wide variety of retroviral vectors can be used, provided that they can be limited to a single round of replication. In addition to sequencing, mutations can be identified in viral genes by biochemical assays, including heteroduplex tracking (HTA), RNase T1 fingerprinting, and detection of single-strand conformation polymorphisms (SSCP). In HTA [109], viral RNA is annealed to a radiolabeled probe and subjected to denaturing-gradient gel electrophoresis. Mismatches caused by viral mutations, particularly in low-melting regions, can alter the thermodynamic stability of the heteroduplex and lead to altered migration during electrophoresis. For RNase T1 fingerprinting [110], radiolabeled viral RNA is digested with RNase T1, which cleaves at G residues in single-stranded RNA. The digested viral RNA is subjected to two-dimensional electrophoresis-homochromatography, generating a map, or fingerprint, of RNase T1-resistant oligonucleotides. Mutations within the larger RNase T1-resistant oligonucleotides often alter migration, allowing for their detection. Lastly, in SSCP [12], PCR of viral genes is performed from infected cell clones. The PCR products are radiolabeled, denatured, and analyzed by non-denaturing gel electrophoresis. Again, mutations are detected by altered mobility during electrophoresis. The primary drawback of these biochemical approaches is that they are labor-intensive, and are thus not conducive to identifying and characterizing large numbers of viral mutants. Additionally, they cannot detect all mutations (RNase T1 fingerprinting and SSCP have been estimated to detect ~80% of changes) [12,110], and thus calculated mutation rates underestimate true mutation rates.

In place of biochemical assays, other groups have simply amplified viral genes by PCR and identified mutations by Sanger sequencing [111,112]. In this instance, nearly all mutations can be identified, provided that they do not prevent amplification. Unfortunately, like biochemical assays, this approach is extremely low-throughput. For example, assuming a mutation rate of 3 × 10^−5^ mutations/bp/cycle (the retroviral average) and a target size of ~800 bp (the length of a typical Sanger read), ~0.024 mutations will be identified per read. Thus, in order to obtain 50 mutations, ~2000 Sanger reads would be required. Accordingly, studies following this approach have reported very low numbers of mutations, which hinders a robust determination of mutation rates and spectra. Additionally, without enriching for mutated sequences, the presence of background errors due to PCR and sequencing becomes a much greater concern, and the necessary controls to account for this must be included. To circumvent the inefficiency of Sanger sequencing, some groups have sequenced virus passaged multiple (~5–10) times [118,119], but mutation rates cannot easily be calculated in these experiments due to purifying selection and uncertainty regarding the number of rounds of replication.

#### 2.3.2. Next-Generation Sequencing-Based Approaches

The advent of next-generation sequencing (NGS) technologies has revolutionized biology, and retrovirology has been no exception. NGS has been used to infer co-receptor usage [120,121], examine immune escape variants [122], and identify minority variants [123,124,125,126] that may contribute to drug resistance in samples from HIV-1-infected individuals. Illumina platforms currently dominate the market due to their high outputs and low costs. Illumina can presently generate ~45–50 million 2 × 300 bp paired-end reads (MiSeq) or ~600 million 2 × 150 bp paired-end reads (HiSeq 2500 in rapid mode). While NGS can identify thousands of variants, NGS technologies are currently hindered by high error rates, due to both PCR and sequencing. Although sequencing libraries can be created in the absence of amplification, PCR is often employed to generate more material for sequencing and to enrich for adapter-ligated templates. The background error rates from NGS have been estimated to range from 10^−2^ mutations/bp for unprocessed data to 10^−3^ or 10^−4^ mutations/bp for processed data [127,128,129,130,131]. Given this, the average retroviral mutation rate is minimally ~10 fold lower than the background error rate, which prevents distinction of biological mutations from background errors. This problem is illustrated by a recent study [40] in which HIV-1 was allowed to replicate for a single cycle in primary CD4^+^ T-cells, amplified by PCR, and subjected to 454 sequencing. The background error rate was determined by amplifying and sequencing plasmid DNA and found to be 7.4 × 10^−5^ mutations/bp, while the error rate in the biological sample was found to be 1.2 × 10^−4^ mutations/bp. Thus, assuming equivalent background levels, ~60% of the mutations were artifactual, and these mutations could not be distinguished from those induced during the viral life cycle. Nonetheless, the mutation rate of HIV-1 was estimated by subtracting the background error rate from the error rate of the biological sample, leading to an estimate of ~4.6 × 10^−5^ mutations/bp/cycle. Recently, two key strategies have been developed to help circumvent the high error rates of NGS. In the first strategy, which has been referred to as low-error amplicon sequencing (LEA-Seq) [132], unique identifier tags (UIDs), such as twelve random bases, are assigned to each template molecule, either through ligation, reverse transcription, or linear PCR [125,132,133,134,135]. Next, exponential PCR of the tagged molecules is performed, followed by redundant sequencing. Consensus families can be built from sequences with identical tags, allowing the identification and exclusion of most PCR and sequencing errors. LEA-Seq has been used for 16S rRNA sequencing in microbial metagenomics [132,134], identification of mutations in human mitochondrial and nuclear DNA [133,135], determination of DNA polymerase fidelity during PCR [135], and tracking minority variants in the HIV-1 protease during drug exposure [125]. In the second strategy, called circular resequencing (CirSeq), rolling circle amplification of template molecules is used to generate three copies from the same starting template molecule that are linked in tandem [136,137,138]. After the linked copies are sequenced, consensus families are again built, permitting the removal of most background errors. CirSeq was recently used to determine the mutation rate and spectrum of poliovirus, as well as to track the fitness of poliovirus variants across serially passaged populations [138]. While neither method has yet been used to determine mutation rates or spectra of retroviruses, both methods are likely to advance our understanding of how mutations arise in viral genes during retroviral replication. 

## 3. Vectors for Examination of Retroviral Recombination

### 3.1. Reporter Gene Vectors

#### 3.1.1. Antibiotic Resistance Genes

**Figure 2:**
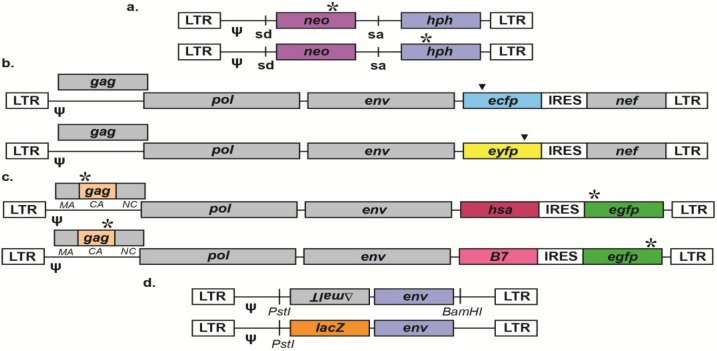
Vector pairs for the investigation of retroviral recombination. Most accessory genes and cis elements are not shown for simplicity; * represents inactivating mutations, while ψ represents packaging signals. (**a**) SNV vector pair that detects recombination events that restore *neo* and *hph*, resulting in viruses that confer G-418 and hygromycin B double drug-resistance to infected cells [43]; sd = splice donor, sa = splice acceptor. (**b**) HIV-1 vector pair that detects recombination events between *ecfp* and *eyfp*, which leads to expression of a modified GFP [55]; ▼ = essential mutations in *ecfp* and *eyfp*. (**c**) HIV-1 vector pair that can simultaneously detect recombination events that restore the capsid (CA) portion of the Gag polyprotein (by staining with an appropriate antibody) and enhanced green fluorescent protein (EGFP) [71]. Viral infection is monitored with cell surface markers like the mouse heat stable antigen (HSA) and B7; IRES = internal ribosome entry site. (**d**) HIV-1 vector pair that detects recombination events that occur within a region of homology (such as the viral env) inserted between β-galactosidase (lacZ) and a BamHI restriction site [77].

Similar to the initial vectors used to investigate retroviral mutation rates, the first vectors used to quantify a retroviral recombination rate relied upon a cassette containing two antibiotic resistance genes (*neo* and *hph*) in an SNV-derived vector (see Table 2 and Figure 2A) [43]. Two similar vectors were engineered: one with a dysfunctional *neo* gene, and the other with a dysfunctional *hph* gene. In both cases, the respective antibiotic resistance genes were disrupted by 4-bp insertions (~1 kb apart), which were found to spontaneously revert at low rates. To examine recombination, dually-infected producer cell clones were created by sequential infection with the parental viruses at low multiplicities of infection (MOI). PCR and enzyme digestion were performed to ensure that producer cell clones contained a single copy of each parental virus and that recombination did not occur during the generation of producer cell clones. While more laborious than simply co-transfecting viral vectors, this procedure eliminates transfection-induced recombination and more closely mimics natural viral replication. Viruses were collected from producer cell clones and used to infect target cells at low MOIs. The recombination rate was determined by dividing the titer of double drug-resistant (neo^r^/hygro^r^) colonies by the lower of the two single drug-resistant titers. Using this method, the recombination rate of SNV was found to be ~2%/kilobase/cycle [43]. Similar MLV-based vectors were later devised and used to show that recombination rates increase with the distance between genetic markers [139]. Additionally, MLV-based vectors have been used to characterize the effects of moving the dimerization linkage structure on recombination rates [140]. More recently, HIV-1-based *neo*/*hph* vectors were used to demonstrate that the HIV-1 recombination rate is ~10-fold higher than in gammaretroviruses [41]. Notably, experiments with these vectors must be performed at low MOIs, as co-infection can produce the same phenotype as a recombinant. Often, Southern blotting of recombinants is required to definitively demonstrate that double-resistant cell clones are not due to co-infection. In addition, this system is not easily amenable to other cell lines and primary cell types, as antibiotic sensitivities can vary widely between cell types.

**Table 2:** Targets for analysis of retroviral recombination.

Target^1^	Assay Type^2^	Virus	References
***neo*/*hph***	Reporter Gene	SNV, MLV, HIV-1	[41,43,139,140,141,142,143]
***tk***	Reporter Gene	MLV	[144]
***lacZ***	Reporter Gene	MLV, HIV-1	[42]
***gfp***	Reporter Gene	MLV, HIV-1, HIV-2, SIV_agm_	[38,49,68,69,70,71,145,146,147]
***ecfp/eyfp***	Reporter Gene	HIV-1	[55]
**viral**	Gag Reconstitution	HIV-1	[71]
	Modified LacZ	HIV-1	[77,148,149]
	PCR/qPCR	HIV-1	[148,150,151,152]
	HTA	HIV-1	[36,37,152]
	Restriction Mapping	HIV-1	[37,45]
	Sequencing (Sanger)	HIV-1	[37,39,45,151]
	Sequencing (NGS)	HIV-1	[40,153]

^1^ Reporter gene targets include antibiotic resistance genes (*neo*, *hph*), thymidine kinase (*tk*), β-galactosidase (*lacZ*), and fluorescent proteins (*gfp*, *ecfp*, *eyfp*); ^2^Reporter gene assays typically detect recombination events that rescue defective gene products. Other assays have been used to detect recombination events in viral genes, such as a Gag reconstitution assay (in which Gag itself is treated as a reporter gene), a modified LacZ assay, PCR or quantitative PCR, heteroduplex tracking (HTA), restriction digest mapping, Sanger sequencing, and next-generation sequencing (NGS) technologies.

#### 3.1.2. Fluorescent Proteins

Retroviral recombination rates have often been measured with fluorescent proteins (Table 2), eliminating the need for antibiotic selection of target cells. Importantly, these vectors facilitate the analysis of recombination in a variety of cell types (including primary cells), as the antibiotic sensitivities of different cell lines do not need to be considered. In one vector system that has often been used for HIV-1, recombination rates are measured using combinations of vectors with distinct inactivating mutations in *gfp* [38]. Specifically, these vectors contain cassettes encoding a cell surface marker (HSA or Thy-1) used to track infectivity, an IRES element, and an inactivated GFP as the recombination target. The expression of GFP can be eliminated by introducing mutations at various positions allowing for measurement of recombination at distances ranging from ~100 to 600 bp. To determine recombination rates, producer cell clones were first created by sequential infection at low MOIs (0.05–0.1). Cell sorting of infected cells was used to purify dually infected producer cells, which express both cell surface markers (HSA and Thy-1). Virus production from producer cells was initiated by the transfection of helper plasmids, and collected viruses were used to infect target cells. Recombination rates in target cells were calculated by comparing the frequency of GFP^+^ cells to all infected cells (HSA^+^ and/or Thy-1^+^). As the experiments were performed at relatively high MOIs (0.4–0.5), some cells were very likely co-infected. In contrast to the dual-antibiotic resistance vectors described in Section 3.1.1., co-infection with the inactivated GFP vectors does not result in a recombinant phenotype. However, co-infection does influence the calculation of the total number of infected cells, which in turn could alter calculated recombination rates. Therefore, the numbers of infected cells were converted to MOIs based upon a Poisson distribution of co-infection in order to more accurately estimate recombination rates [38]. In some studies, further analyses were done to confirm recombination events in *gfp* and map recombination breakpoints in viral genes [70,147]. In order to accomplish this, single GFP^+^ cell clones were isolated by cell sorting and propagated independently in order to prevent PCR-mediated recombination between proviruses. PCR and restriction mapping were used to verify reconstitution of functional *gfp* genes, as the inactivating mutations in *gfp* were designed to introduce specific restriction sites. Sanger sequencing of PCR products was used to further confirm recombination in *gfp*. Additional PCR and Sanger sequencing of GFP^+^ cell clones has often been performed to identify crossovers in viral sequences as well. Of note, rather than isolating single cell clones, a modified type of PCR called single-genome sequencing (SGS) has also been used to prevent PCR-mediated recombination [49]. In SGS, templates (e.g., viral RNA or proviral DNA) are diluted such that <30% of reactions result in products. Under these conditions, the vast majority of reactions contain only a single template, thus preventing most PCR-mediated recombination between templates. 

Initially, vectors based on inactivated GFP were used to examine the impact of distance, cell type (primary *vs*. immortalized T-cells), and accessory genes on the recombination rate of HIV-1 [38]. Similar vectors were later used to measure recombination rates in non-B subtypes of HIV-1 as well as rates of intersubtype or intergroup recombination [49,68,69,70,71]. Further, these vectors have been used to study the impact of cell type (T-cells, monocytes, and macrophages) [145] on recombination. In addition to HIV-1, analogous vectors have been engineered for HIV-2 and simian immunodeficiency virus from African green monkeys (SIV_agm_), both of which were found to recombine at similar rates to HIV-1 [146]. These vectors have also been used to demonstrate that HIV-1 and HIV-2 are able to co-package and recombine [147], albeit at very low rates (recombination frequency of 0.2%, ~35-fold lower than for intrasubtype recombination in HIV-1). 

In addition to GFP, several other fluorescent proteins have been used in recombination vectors such as enhanced yellow and cyan fluorescent proteins (EYFP and ECFP). These vectors take advantage of the high level of homology between different fluorescent proteins. Specifically, infectious molecular clones of HIV-1 have been developed that encode EYFP or ECFP in place of the accessory protein Nef, with Nef expressed from a downstream IRES element (see Figure 2B) [55]. As the critical residues in *yfp* and *cfp* are ~400 bp apart, recombination between these residues gives rise to a modified *gfp* gene (called gfp*) that can be spectrally separated from *yfp* with the appropriate filters. Thus, recombination frequencies were calculated by determining the ratio of GFP^+^ to EYFP or ECFP^+^ cells during flow cytometry. To map recombination breakpoints in gfp*, single GFP^+^ cell clones were isolated by FACS, amplified by PCR, and sequenced. Sequencing was also performed to map crossovers in viral genes, which could easily be identified as the vectors were engineered in two distinct isolates of HIV-1. Although these vectors express all viral genes and are replication-competent, replication can still be limited to a single round of replication through use of an antiviral drug. 

#### 3.1.3. LacZ (β-galactosidase)

Recombination rates have occasionally been determined using other reporter genes such as the full-length *lacZ* gene encoding β-galactosidase [42]. For example, HIV-1 and MLV vector pairs have been designed in which one encodes a functional *lacZ* gene but no antibiotic resistance gene, while the other encodes a defective *lacZ* gene and a puromycin resistance gene (*puro*). Recombination rates were calculated by determining the ratio of *lacZ^+^*/puromycin-resistant (puro^r^) colonies (*i.e.*, puro^r^ colonies that stain blue in the presence of X-gal) to all puro^r^ colonies. These vectors have been used to compare frequencies of recombination between MLV and HIV-1, as well as to investigate factors responsible for observed differences between these viruses.

#### 3.1.4. Direct Repeat Reporter Vectors

Directly repeated sequences in retroviral genomes and vectors have long been known to delete at high frequencies during reverse transcription [154,155,156,157]. Deletions occur at high rates regardless of whether the repeats are in tandem or separated by additional sequences. In one of the earliest observations of this phenomenon, the *src* oncogene was found to be frequently lost during RSV replication due to flanking direct repeats [155,156]. Direct repeat deletions also contribute to the variable copy number of repeats that have been observed in the enhancer sequences of retroviral LTRs [158,159]. These deletions have been shown to occur primarily through intramolecular template switching events, rather than through homologous recombination between separate viral genomes [141]. Retroviral vectors have been developed that take advantage of the high efficiency of direct repeat deletion to assay the impact of various factors on template switching. These vectors have large (>100 bp) inactivating duplications in reporter genes, such as *tk*, *lacZ*, or *gfp*. Direct repeat deletion eliminates the duplicated sequence, thus restoring the reporter gene phenotype. In one early study, an MLV vector was engineered with a *tk*/*neo* cassette in which *tk* contained an internal 700 bp duplication [144]. Deletion frequencies were first assessed by comparing the ratio of *tk*^+^ (*i.e.*, aminopterin-resistant)/neo^r^ colonies to all neo^r^ colonies. Southern blotting and densitometry were performed to determine deletion frequencies in a more quantitative fashion. In several other studies, MLV and HIV-1-based vectors were engineered in which *lacZ* reporter genes were inactivated by internal duplications [42,74,80,160,161]. Deletion frequencies were calculated by comparing titers of *lacZ*^+^/puro^r^ cell colonies to all puro^r^ colonies. Lastly, MLV and HIV-1 vectors with internal duplications (of 200–250 bp) in *gfp* have been developed [72,73,75]. With these vectors, direct repeat deletion leads to reconstitution of functional GFP, and deletion frequencies can be determined as the ratio of GFP^+^/antibiotic-resistant cell colonies to all antibiotic-resistant cell colonies. More recently, a similar HIV-1 vector was developed in which the antibiotic resistance gene was replaced with the cell surface marker *hsa*, facilitating use in primary cells [162]. As direct repeat deletion is very efficient during reverse transcription, the ability of various factors to impact deletion frequencies can readily be determined, even when such effects are relatively small. However, as discussed, direct repeat deletions primarily occur through intramolecular template switching events rather than intermolecular template switching events [141]. Thus, the outcomes observed in these studies may not necessarily be representative of retroviral recombination, which requires intermolecular template switching events between distinct viruses. 

### 3.2. Detection of Recombinants in Viral Genes

#### 3.2.1. Gag Reconstitution Assay

Recently, a new HIV-1 vector system has been engineered that is based on the reconstitution of functional Gag protein (Figure 2C) [71]. This vector system combines many of the best features of reporter assays with the increased biological relevance of viral genes. The concept behind the vector system was straightforward: Gag is expressed at high levels on the plasma membranes of infected target cells, such that Gag itself can be treated as a reporter gene with a reliable anti-Gag antibody. More specifically, multiple pairs of vectors were engineered in different subtypes and groups of HIV-1 with distinct inactivating mutations separated by ~300 bp in the capsid portion of the Gag polyprotein. In addition, the *gfp* genes were inactivated in these vectors, allowing for simultaneous detection of recombination in identical (*gfp*) and non-identical (*gag*) target sequences. Cell-surface markers such as HSA and B7 (*i.e.*, human CD80) permitted tracking of viral infectivity. These vectors were sequentially introduced into producer cells, complemented with Gag-Pol in trans to produce infectious virus, and used to infect target cells. Target cells were stained with a PE-conjugated anti-p24 (capsid) antibody to detect recombination events that led to expression of full-length Gag. The authors showed that the particular antibody they used could reliably detect a variety of parental Gag proteins from different subtypes and groups of HIV-1, as well as recombinants between subtypes and groups. Recombination rates were determined by comparing the frequency of Gag^+^ cells to all infected cells (as determined by the cell surface markers). Furthermore, since these vectors also contain defective forms of *gfp*, recombination rates could simultaneously be determined in identical (albeit foreign) gene sequences across viruses. While representing a tremendous improvement over standard reporter gene vectors, these vectors are limited by a small dynamic range of the assay due to the fixed distance (~300 bp of capsid) of the mutations. However, suitable antibodies that recognize other regions of the unprocessed Gag polyprotein may be identified in the future, allowing for analysis of a larger region of Gag. Generally, this approach would likely not be amenable for identifying recombinants in other viral genes and non-coding regions of the genome. Additionally, some Gag recombinants may not be identified if, for example, they prevent proper folding or recognition by the antibody.

#### 3.2.2. Use of LacZ (β-galactosidase) to Detect Recombinants in Viral Genes

Additional retroviral vectors have been developed that take advantage of reporter gene phenotypes but actually detect recombination events within viral targets. Specifically, vector pairs for different groups and subtypes of HIV-1 have been designed that contain either a functional *lacZ* or a completely unrelated sequence in its place (such as a defective *malT* gene in reverse orientation), as demonstrated in Figure 2D [77,148,149]. After the *lacZ* gene, *env* genes from various viral isolates were inserted, followed by a BamHI restriction site (in the *lacZ*^-^ vector) or no restriction site (in the *lacZ*^+^ vector). After PCR amplification, proviral DNA was digested with the BamHI restriction enzyme (in conjunction with PstI), ligated into a cloning vector, and subjected to blue-white color screening. Recombination in *env* results in *lacZ*^+^ proviruses with the BamHI restriction site, which can then efficiently ligate into the cloning vector. The recombination frequency is then computed by determining the ratio of *lacZ*^+^ colonies to all colonies. In this approach, all observed recombination events must have occurred within *env* rather than *lacZ*, as one vector completely lacks the *lacZ* gene. When *env* genes from different viral isolates are used, sequencing can be performed to identify the exact breakpoints. While this approach is able to identify recombinants in viral genes, it is somewhat laborious and involves the placement of viral genes outside of their native sequence contexts.

#### 3.2.3. Heteroduplex Tracking, Quantitative PCR, and Restriction Mapping

Numerous biochemistry and molecular biology-based techniques have been used to directly identify and quantify retroviral recombinants occurring within viral genes, without any assistance from reporter genes. Most often, these assays rely upon the detection of sequence differences between distinct viral isolates, either from the same subtype (intrasubtype) or different subtypes (intersubtype), groups, or types of HIV. Thus, recombination frequencies from these assays may somewhat underestimate recombination rates between highly homologous viruses. Even when these assays are able to detect recombination between nearly identical viruses (by, for example, restriction mapping), the recombination breakpoints cannot be better defined unless silent point mutations have been introduced to create intervening intervals. These types of approaches have been used to identify recombinants in viral genes during both single cycle and multi-cycle infections. In spreading infections, viral recombinants are subject to strong purifying selection, which can restrict observable recombination events. In some cases, parental retroviral vectors have been used that contain different defects in essential viral genes (such as deletions in *pol* and *env*), such that only recombinants are able to spread after the initial round of replication [49,163,164]. 

In several reports, heteroduplex tracking (HTA) has been performed to identify recombinants in viral genes [36,37,152], which can detect recombinants (as well as mutants) when there are sufficient sequence differences between the parental viruses. Recombination can alter the affinity of a viral sequence for a radioactive probe (relative to the two parental DNAs), which can be detected as a shift in band pattern during gel electrophoresis. HTA has been estimated to reliably detect sequence divergence when the mismatches exceed ~1% [165,166], and thus this technique can only identify crossovers introducing >1% mismatch relative to the parental viruses. Therefore, some recombinants cannot be detected by this method, particularly when the parental isolates are quite similar (e.g., from the same HIV-1 subtype). This method also requires sequencing of fragments to confirm that gel shifts were not caused by mutations, insertions, or deletions. Restriction mapping, Southern blotting (with short oligonucleotides specific to one of the parental isolates), and/or Sanger sequencing can be used to further refine recombination breakpoints between the viruses. 

As an alternative to HTA, several studies have used PCR or restriction mapping to detect recombination [37,45,148,150,151,152]. PCR has been performed using primers that are specific to one of the two parental viruses. By combining one primer from each virus, recombinants can be detected. Additionally, different forms of quantitative PCR have been used to determine starting amounts of recombinant and parental DNAs, enabling calculation of recombination frequencies. As for HTA, Southern blotting or Sanger sequencing can be used to more precisely identify crossover points between distinct viral isolates. These approaches have been used to track HIV-1 intersubtype viral recombinants, and have led to identification of recombination hotspots as well as characterization of the potential mutagenicity of recombination.

#### 3.2.4. Direct Sequencing of Viral Genes-Sanger

As discussed above, Sanger sequencing of viral genes has often been employed to further characterize recombinants identified by techniques such as HTA, PCR, or restriction mapping. Sanger sequencing has often also been used to find crossovers in viral genes from proviruses that are recombinants in reporter genes. In addition, recombinants have been identified simply by direct amplification and sequencing of viral genes from infected cells [37,39,151]. The low-throughput nature of direct Sanger sequencing is somewhat less problematic for studies of retroviral recombination than for mutagenesis. This is because some retroviruses, such as lentiviruses, recombine ~10 times more frequently than they mutate. While sequencing is often used to map recombination breakpoints between heterogeneous viruses, HIV-1 vectors have recently been engineered that allow detection of recombination between highly homologous viruses, thus more accurately representing the viral population found within infected individuals [39]. Specifically, silent mutations that could be detected by PCR and Sanger sequencing were introduced throughout the *gag* gene of an HIV-1 infectious molecular clone. These silent mutations served as markers for recombination and did not alter the protein sequences or any known cis elements. Further, they were shown to have no effect on viral infectivity. Most markers actually consisted of two mutations, typically spaced three bases apart, to facilitate differentiation of markers from mutations or background errors. Notably, the vectors they designed encode functional open reading frames for all viral proteins. In order to investigate HIV-1 recombination, viral stocks were first produced by co-transfecting the wild-type and marker plasmids into 293T cells. This is in contrast to many earlier studies in which producer cell clones were generated by sequential infection or, in some cases, co-infection with parental viruses [38,41,43,68,69,70,139,140,141,142,143,145,146,147]. While sequential infection is more time-consuming, it eliminates the possibility of transfection-induced recombination (TIR) and more closely resembles native viral replication. However, the investigators of this study measured the frequency of TIR by direct sequencing of plasmid DNA from co-transfected cells and found that the frequency of TIR was much lower than the recombination rates measured for HIV-1. Next, the authors collected virus from co-transfected cells, infected primary T-cells, performed PCR, and identified recombinants by Sanger sequencing. Virus replication was limited to a single round using the T-20 fusion inhibitor. All PCR reactions were terminated in the log-linear phase of amplification, which reduces the frequency of PCR-mediated recombination [167]. Additional controls were performed to show that PCR-induced and intervirion recombination did not substantially contribute to the observed data. Further, the authors estimated the fraction of heterozygous virions directly from the sequencing data and developed a mathematical model that accounted for the possibility of multiple template switches, based on distances between genetic markers and observed recombination rates. Using this vector system, the HIV-1 recombination rate was observed to be 0.81 × 10^−3^ recombination events per nucleotide (REPN), corresponding to a true recombination rate of 1.35 × 10^−3^ REPN (or 12.5 crossovers/genome) after considering the possibility of multiple template switches. 

#### 3.2.5. Direct Sequencing of Viral Genes-NGS

In several subsequent studies, similar HIV-1 vectors with marker mutations throughout the *gag* and *pol* genes have been applied to 454 sequencing [40,153]. As described above, wild-type and marker vectors were co-transfected into 293T cells, and viruses were collected and used to infect primary blood mononuclear cells. Although replication-competent, the virus was limited to a single round of replication with fusion inhibitor. Proviral DNA was amplified by PCR, sequencing libraries were prepared, and 454 sequencing was performed, generating ~1 million reads per run. Experimental sources of recombination, such as transfection-induced recombination, intervirion recombination, and PCR, were carefully measured in parallel. These background sources were found to result in recombination rates less than 10% of the recombination rate measured in the biological samples. Using these vectors and 454 sequencing, the overall rates of recombination in HIV-1 *gag* and *pol* were found to be 1.5–2 × 10^−3^ REPN, or ~14–19 crossovers per genome. In addition, using the large amounts of data generated by 454, recombination hot and cold spots could be identified in viral genes [153]. Further, ~15%–20% of mutations were found to be associated with recombination, though the direction of causality could not be determined [40]. As the association of recombination and mutation is itself a topic of great research interest, the high background error rates of NGS technologies are also somewhat problematic for studies of viral recombination. Thus, recent improvements aimed at lowering NGS error rates (see Section 2.3.2.) will surely benefit recombination studies as well. Additionally, these improvements often permit the identification and exclusion of PCR-induced crossovers as well as errors, thus limiting the effects of PCR-mediated recombination on the observed data.

## 4. Conclusions

Given the wide variety of retroviral vectors available, choosing a particular vector to assess retroviral mutagenesis and/or recombination can be daunting. Retroviral vectors based on reporter genes offer a number of distinct benefits that have led to their widespread use in mutation and recombination studies. First, mutants or recombinants can be efficiently detected based on phenotypes that are easily observed and quantified, while mutants or recombinants in viral targets must be detected through much more laborious means. Targeted sequencing of mutant viruses can determine the nature of the underlying mutations, while targeted sequencing of recombinants can confirm that recombination took place (though the breakpoint locations cannot be precisely defined). In addition, reporter gene vectors are generally somewhat less dependent on PCR for identification of mutants or recombinants, and thus relatively less impeded by PCR-induced errors or crossovers. Nonetheless, reporter gene-based approaches are limited in that they may not necessarily be reflective of viral gene sequences. Large amounts of evidence have shown that patterns of mutation and recombination are affected by factors like homopolymeric runs and secondary structures [77,78,79,92,93,94,95,152], which may vary between template sequences. Given this, the degree to which reporter genes accurately represent viral genes cannot be easily ascertained at present, as so few studies have examined large numbers of mutations or crossovers in viral genes. Additionally, reporter gene vectors typically do not express all viral genes, though some exceptions do exist. In contrast, native viral genes are viewed as more desirable targets of mutation and recombination, but detection of mutation or recombination is often very laborious and time-consuming. Next-generation sequencing (NGS) technologies offer an obvious solution to these barriers, but standard NGS methods result in high background error rates, hindering identification of true mutations. Additionally, NGS is heavily reliant on PCR for sample preparation, which, under standard conditions, generates high levels of artifactual mutations and recombination events. However, several strategies for NGS sample preparation have recently been established that allow for identification and exclusion of the vast majority of background errors and crossovers due to PCR or sequencing [125,133,135,136,138]. While these methods have not yet been used to quantify retroviral mutation or recombination rates, they will be key to advancing our understanding of retroviral mutagenesis and recombination in viral genes. In the near future, standardized retroviral protocols, vectors, and assays will hopefully be developed that allow for application of these improvements to a wide variety of questions in retrovirology. 
